# Supplementary material for: Do women prefer caesarean sections? A qualitative evidence synthesis of their views and experiences
Source: PLoS One. 2021 May 5;16(5):e0251072. doi: 10.1371/journal.pone.0251072 (PMC8099111; doi:10.1371/journal.pone.0251072)
Supplement: S1 Table — (DOCX) [file pone.0251072.s001.docx]

**S1. Search strategies**

**Initial searches December 2016**

*PubMed / Medline*

*http://www.pubmed.gov*

| # | Searches | Results |
| --- | --- | --- |
| 1 | "Cesarean Section"[Mesh] OR Cesarean [TIAB] OR Caesarean [TIAB] OR “C-sections” [TIAB] OR “C-section”[TIAB] OR “Abdominal Deliveries”[TIAB] OR “Abdominal Delivery”[TIAB] OR Postcesarean [TIAB] OR Postcaesarean [TIAB] OR “Post cesarean” [TIAB] OR “Post caesarean” [TIAB] | 59563 |
| 2 | "Patient Preference"[Mesh] OR "Nurse-Patient Relations"[Mesh] OR "Interviews as Topic"[Mesh] OR "Emotions"[Mesh] OR "Elective Surgical Procedures"[Mesh] OR "Unnecessary Procedures"[Mesh] OR "Physician-Patient Relations"[Mesh] OR "Patient Satisfaction"[Mesh] OR "Natural Childbirth"[Mesh] OR "Health Knowledge, Attitudes, Practice"[Mesh] OR "Choice Behavior"[Mesh] OR "Attitude to Health"[Mesh] OR "Cesarean Section/psychology"[Mesh] OR "Culture"[Mesh] OR "ethnology" [Subheading] OR "Vaginal Birth after Cesarean"[Mesh] OR “decision making” [MH] OR “pain/psychology” [MH] OR “Delivery, obstetric/psychology” [MH] OR “pregnant women/psychology” [MH] OR Patient Participation[Mesh] OR Fear* [TIAB] OR preference* [TIAB] OR prefers [TIAB] OR preferred [TIAB] OR decision* [TIAB] OR “non medical factors” [TIAB] OR “non medical indications” [TIAB] OR option [TIAB] OR options [TIAB] OR optional [TIAB] | 1520832 |
| 3 | Step 1 and Step 2 | 7793 |
| 4 | Publication date from 1990/01/01 to 2016/12/31 | 7191 |

*Note: We set the search date considering the rapid increase of CS rate over the past three decades worldwide. We follow international literature on this topic which usually focus on data from 1990 to assess trends. Recent WHO estimates on trends for caesarean section rates reported data from 1990 as an approximate reasonable date for the unprecedented increase of CS use.*

*PsycINFO*

*http://search.ebsco.com*

| # | Searches | Results |
| --- | --- | --- |
| 1 | DE "Caesarean Birth" OR TI Cesarean OR TI Caesarean OR TI (C-sections) OR TI(C-section) OR TI (Abdominal Deliveries) OR TI (Abdominal Delivery) OR TI Postcesarean OR TI Postcaesarean OR TI (Post cesarean) OR TI (Post caesarean) OR AB Cesarean OR AB Caesarean OR AB (C-sections) OR AB (C-section) OR AB (Abdominal Deliveries) OR AB (Abdominal Delivery) OR AB Postcesarean OR AB Postcaesarean OR AB (Post cesarean) OR AB (Post caesarean) | NA |
| 2 | DE "Preferences" OR DE "Decision Making" OR DE "Emotional States" OR TI Fear* OR TI preference* OR TI prefers OR TI preferred OR TI decision* OR TI (non medical factors)   OR TI (non  medical indications) OR TI option  OR TI options  OR TI optional OR AB Fear* OR AB preference* OR AB prefers OR AB preferred OR AB decision* OR AB (non medical factors)   OR AB (non  medical indications) OR AB option  OR AB options  OR TI optional | NA |
| 3 | Published Date: 1990/01/01-2016/12/31 | 269 |

*CINAHL*

*http://search.ebsco.com*

| # | Searches | Results |
| --- | --- | --- |
| 1 | (MH "Cesarean Section+") OR TI Cesarean OR TI Caesarean OR TI (C-sections) OR TI(C-section) OR TI (Abdominal Deliveries) OR TI (Abdominal Delivery) OR TI Postcesarean OR TI Postcaesarean OR TI (Post cesarean) OR TI (Post caesarean) OR AB Cesarean OR AB Caesarean OR AB (C-sections) OR AB (C-section) OR AB (Abdominal Deliveries) OR AB (Abdominal Delivery) OR AB Postcesarean OR AB Postcaesarean OR AB (Post cesarean) OR AB (Post caesarean) | NA |
| 2 | (MH "Professional-Patient Relations+") OR (MH "Interviews+") OR (MH "Surgery, Elective+") OR (MH "Unnecessary Procedures") OR (MH "Attitude to Health+") OR (MH "Alternative Birth Methods+") OR (MH "Home Childbirth") OR (MH "Prepared Childbirth") OR (MH "Culture+") OR (MH "Ethnological Research") OR (MH "Vaginal Birth+") OR (MH "Health Knowledge") OR (MH "Decision Making, Patient") OR (MH "Decision Support Techniques") OR (MH "Decision Making, Family") OR (MH "Pain/PF") OR (MH "Expectant Mothers/PF") OR (MH "Delivery, Obstetric/PF") OR TI Fear* OR TI preference* OR TI prefers OR TI preferred OR TI decision* OR TI (non medical factors)   OR TI (non  medical indications) OR TI option  OR TI options  OR TI optional OR AB Fear* OR AB preference* OR AB prefers OR AB preferred OR AB decision* OR AB (non medical factors)   OR AB (non  medical indications) OR AB option  OR AB options  OR TI optional | NA |
| 3 | Published Date: 1990/01/01-2016/12/31 | NA |
| 4 | Exclude MEDLINE records | 356 |

*EMBASE*

*http://www.embase.com*

| # | Searches | Results |
| --- | --- | --- |
| 1 | 'cesarean section'/exp OR 'cesarean section' OR 'cesarean section kit'/exp OR 'cesarean section kit' OR cesarean:de,ab,ti OR caesarean:de,ab,ti OR 'c-sections':de,ab,ti OR 'c-section':de,ab,ti OR 'abdominal deliveries':de,ab,ti OR 'abdominal delivery':de,ab,ti OR postcesarean:de,ab,ti OR postcaesarean:de,ab,ti OR 'post cesarean':de,ab,ti OR 'post caesarean':de,ab,ti | 90871 |
| 2 | 'patient preference'/exp OR 'nurse patient relationship'/exp OR 'interview'/exp OR 'emotion'/exp OR 'elective surgery'/exp OR 'unnecessary procedure'/exp OR 'doctor patient relation'/exp OR 'patient satisfaction'/exp OR 'natural childbirth'/exp OR 'attitude to health'/exp OR 'cultural anthropology'/exp OR 'ethnology'/exp OR 'patient participation'/exp OR fear*:de,ab,ti OR preference*:de,ab,ti OR prefers:de,ab,ti OR preferred:de,ab,ti OR decision*:de,ab,ti OR 'non medical factors':de,ab,ti OR 'non medical indications':de,ab,ti OR option:de,ab,ti OR options:de,ab,ti OR optional:de,ab,ti OR (('vaginal birth' OR 'vaginal births') NEAR/2 (cesarean OR caesarean)):de,ab,ti OR ('pregnant woman' NEAR/10 psycholog*):de | 1936849 |
| 3 | Step 1 and Step 2 | 12479 |
| 4 | [1990-2016]/py | 11753 |

*Popline*

[*http://www.popline.org*](http://www.popline.org)

| # | Searches | Results |
| --- | --- | --- |
| 1 | ((Cesarean ) OR (Caesarean ) OR (Cesareans ) OR (Caesareans ) OR (C-sections) OR (C-section) OR (Abdominal Deliveries) OR (Abdominal Delivery) OR (Postcesarean) OR (Postcaesarean)) AND ((Fear) OR (FEARS) OR ( preference) OR (preferences) OR (prefers) OR (prefer) OR (preferred) OR (decision) OR (decisions) OR (non medical factors)   OR (non  medical indications) OR (option)  OR ( options) OR (optional)) | NA |
| 2 | Published Date: 1990-2016 | 414 |

*Global Health Library - Global Index Medicus*

[*http://www.globalhealthlibrary.net/php/index.php*](http://www.globalhealthlibrary.net/php/index.php)

*(Indexes - Regional Indexs) Option selected.*

| # | Searches | Results |
| --- | --- | --- |
| 1 | ((Cesarean ) OR (Caesarean ) OR (Cesareans ) OR (Caesareans ) OR (C-sections) OR (C-section) OR (Abdominal Deliveries) OR (Abdominal Delivery) OR (Postcesarean) OR (Postcaesarean)) AND ((Fear) OR (FEARS) OR ( preference) OR (preferences) OR (prefers) OR (prefer) OR (preferred) OR (decision) OR (decisions) OR (non medical factors)   OR (non  medical indications) OR (option)  OR ( options) OR (optional)) | NA |
| 2 | Published Date: 1990-2016 | 251 |

**Summary of search results**

**Initial searches December 2016**

*Publication date from 01/01/1990 to 31/12/2016*

| Database | Results |
| --- | --- |
| PubMed / Medline | 7191 |
| PsycINFO | 269 |
| CINAHL | 356 |
| EMBASE | 11753 |
| Popline | 414 |
| Global Health Library - Global Index Medicus | 251 |
| Total | 20234 |

**Search update May 2019**

*Publication date from 1/1/2017 to date 31/05/2019*

| Database | Results |
| --- | --- |
| PubMed / Medline | 1572 |
| PsycINFO | 74 |
| CINAHL | 506 |
| EMBASE | 4749 |
| Popline | 55 |
| Global Health Library - Global Index Medicus | 89 |
| Total | 7045 |

**Search update February 2021**

*Publication date from 01/05/2019 to 09/02/2021*

| Database | Results |
| --- | --- |
| PubMed / Medline | 1107 |
