# Supplementary material for: Do women prefer caesarean sections? A qualitative evidence synthesis of their views and experiences
Source: PLoS One. 2021 May 5;16(5):e0251072. doi: 10.1371/journal.pone.0251072 (PMC8099111; doi:10.1371/journal.pone.0251072)
Supplement: S2 Table — (DOCX) [file pone.0251072.s002.docx]

S2 Table. Summary of data analysis and synthesis process

Thematic synthesis utilized comprised four steps:

1. **Familiarization**

Forty-six studies met the review inclusion criteria and were included in the synthesis. We read and re-read the studies to familiarize ourselves with the phenomena of interest explored and study findings.

1. **Data extraction**

The characteristics of included studies, author interpretation (themes, theories and metaphors) and verbatim text (participant quotes) were entered into a data extraction form designed for the purposes of this review.

1. **Coding and development of descriptive themes**

We created an inductively developed codebook with codes initially structured as “free” codes on the basis of the data from identified themes. Codes were updated and refined through comparison with the findings from all included studies and agreed by consensus. All extracted data were then coded into concepts inductively developed following discussion among review author team.

1. **Interpretive synthesis**

Analytical discussions were conducted on the emergent concepts and final interpretive themes generated using a constant comparison strategy, first by reciprocal analysis (confirmatory of similarities in data across studies) and then by refutational analysis (integration of disconfirmatory data into emergent themes). This was a cyclical process and was repeated until the themes generated were sufficiently conceptual to explain and describe the initial descriptive themes from stage 3. Final interpretive themes were then synthesized into Summary of Findings statements.
