# Supplementary material for: Do women prefer caesarean sections? A qualitative evidence synthesis of their views and experiences
Source: PLoS One. 2021 May 5;16(5):e0251072. doi: 10.1371/journal.pone.0251072 (PMC8099111; doi:10.1371/journal.pone.0251072)
Supplement: S3 Table — (DOCX) [file pone.0251072.s003.docx]

**S3.** Assessment of methodological limitations

| Study ID | Was there a clear statement of the aims of the research? | Is a qualitative methodology appropriate? | Was the research design appropriate to address the aims of the research? | Was the recruitment strategy appropriate to the aims of the research? | Were the data collected in a way that addressed the research issue? | Relationship between the researcher and participants been adequately considered? | Have ethical issues been taken into consideration? | Was the data analysis sufficiently rigorous? | Is there a clear statement of findings? | How valuable is the research | Overall assessment of methodological limitations |
| --- | --- | --- | --- | --- | --- | --- | --- | --- | --- | --- | --- |
| Martin 2013 | yes | yes | partial | partial | yes | no | yes | yes | yes | partial | moderate concerns |
| McAra-Coupe 2010 | no | partial | yes | no | yes | yes | yes | yes | partial | partial | major concerns |
| Farnworth 2007 | yes | yes | yes | yes | yes | yes | yes | partial | yes | yes | minor to moderate concerns |
| Nilsson 2017 | yes | yes | yes | partial | yes | no | yes | yes | yes | partial | minor concerns |
| Philips 2009 | yes | yes | partial | partial | yes | partial | yes | partial | yes | yes | moderate concerns |
| Munro 2016 | yes | yes | yes | yes | yes | partial | yes | yes | yes | yes | minor concerns |
| Schantz 2017 | yes | yes | yes | partial | partial | no | yes | partial | yes | partial | moderate concerns |
| Munro 2009 | yes | yes | yes | yes | yes | partial | yes | yes | yes | partial | minor concerns |
| McGrath 2009 | yes | yes | yes | yes | yes | yes | yes | yes | yes | yes | minor concerns |
| Kingdon 2009 | yes | yes | yes | yes | yes | no | yes | yes | yes | yes | minor concerns |
| Sercekus 2009 | yes | yes | partial | no | yes | no | yes | yes | yes | yes | moderate concerns |
| Goodall 2009 | yes | yes | no | partial | yes | yes | partial | yes | yes | yes | minor to moderate concerns |
| Matthias 2009 | yes | yes | yes | yes | yes | yes | yes | partial | yes | partial | moderate concerns |
| Kabakian 2007 | yes | yes | partial | partial | yes | no | no | no | yes | partial | minor to moderate concerns |
| Foureur 2016 | yes | partial | partial | yes | yes | partial | yes | yes | yes | partial | minor concerns |
| Fleming 2017 | yes | yes | yes | no | yes | yes | yes | yes | partial | partial | minor concerns |
| Weaver 2007 | yes | yes | yes | yes | yes | no | yes | yes | yes | partial | minor concerns |
| Moffat 2007 | yes | yes | yes | yes | yes | no | yes | yes | yes | yes | minor concerns |
| Meddings 2007 | yes | yes | yes | yes | yes | no | yes | yes | partial | partial | moderate concerns |
| Liamputtong 2005 | partial | yes | no | yes | yes | no | yes | partial | partial | partial | moderate concerns |
| McCallum 2005 | no | no | no | no | no | no | no | no | partial | no | major concerns |
| Rahnama 2016 | yes | yes | yes | partial | yes | yes | yes | yes | yes | partial | minor concerns |
| Boz 2016 | yes | yes | yes | partial | yes | no | no | yes | yes | partial | moderate concerns |
| Happel 2015 | yes | yes | yes | yes | partial | no | yes | yes | yes | partial | moderate concerns |
| Rahnama 2015 | yes | yes | no | yes | yes | no | partial | yes | partial | partial | minor concerns |
| Shorten 2014 | yes | yes | yes | partial | yes | no | yes | yes | yes | yes | moderate concerns |
| Richard 2014 | yes | yes | no | partial | yes | yes | yes | partial | yes | yes | moderate concerns |
| Johansson 2014 | yes | yes | no | yes | yes | no | yes | yes | yes | yes | minor concerns |
| Chadwick 2014 | yes | yes | partial | yes | no | yes | no | no | yes | yes | moderate to major concerns |
| Kennedy 2013 | yes | yes | no | partial | yes | no | yes | no | yes | yes | moderate concerns |
| Sahlin 2013 | yes | yes | yes | yes | yes | yes | yes | yes | yes | yes | minor concerns |
| Hannah 2013 | yes | yes | yes | yes | yes | no | yes | yes | yes | yes | minor concerns |
| Liu 2013 | yes | yes | yes | yes | yes | no | yes | no | yes | yes | moderate concerns |
| Kabakian 2013 | yes | yes | yes | yes | yes | yes | yes | yes | yes | yes | no concerns |
| Sapountzi 2011 | yes | yes | no | yes | yes | no | yes | yes | yes | partial | minor concerns |
| Douche’a and Carryerb 2011 | partial | yes | no | no | yes | no | yes | no | yes | yes | moderate concerns |
| Kornelsen 2010 | partial | yes | yes | yes | yes | partial | yes | no | yes | partial | minor concerns |
| Behruzi 2010 | yes | yes | yes | partial | yes | yes | yes | yes | yes | yes | minor concerns |
| Pereira 2011 | yes | yes | no | no | yes | no | yes | partial | yes | partial | major concerns |
| Lino 2015 | yes | yes | partial | partial | yes | partial | yes | partial | yes | partial | major concerns |
| Keegan 2014 | yes | yes | yes | yes | yes | yes | partial | yes | yes | yes | minor concerns |
| Chadwick 2013 | yes | yes | partial | yes | partial | no | yes | yes | yes | partial | major concerns |
| Malacrida 2012 | no | yes | partial | partial | partial | no | no | yes | yes | yes | major concerns |
| Latifnejad 2015 | yes | yes | yes | partial | yes | yes | yes | yes | yes | yes | no concerns |
| Litorp 2015 | yes | yes | yes | yes | yes | yes | yes | yes | yes | yes | no concerns |
| Abbaspoor 2014 | yes | yes | partial | partial | yes | yes | yes | yes | yes | partial | minor to moderate concerns |
| Suwanrath 2021 | yes | yes | yes | yes | yes | no | yes | yes | yes | yes | no concerns |
| Waniala 2020 | yes | yes | yes | yes | yes | no | yes | yes | yes | yes | no concerns |
| Husby 2019 | yes | yes | yes | yes | yes | no | yes | partial | partial | partial | minor concerns |
| Schantz 2021 | yes | yes | yes | yes | yes | yes | yes | yes | yes | yes | no concerns |
| Takegata 2020 | yes | yes | yes | yes | yes | no | yes | yes | yes | yes | no concerns |
| Hatamleha 2019 | yes | yes | yes | yes | yes | no | yes | yes | yes | yes | no concerns |
