# Supplementary material for: Do women prefer caesarean sections? A qualitative evidence synthesis of their views and experiences
Source: PLoS One. 2021 May 5;16(5):e0251072. doi: 10.1371/journal.pone.0251072 (PMC8099111; doi:10.1371/journal.pone.0251072)
Supplement: S4 Table — (DOCX) [file pone.0251072.s004.docx]

**S4**. Evidence profile.

| **Review finding** | **Assessment of Methodological limitations** | **Assessment of Relevance** | **Assessment of Coherence** | **Assessment of Adequacy** | **Overall CERQual assessment of Confidence** |
| --- | --- | --- | --- | --- | --- |
| **Deep rooted fears regarding vaginal birth** | **Minor concerns:** There were eight studies (of 27 studies) with major methodological limitations, three studies with moderate concerns, 11 studies with minor concerns including limited description of sampling, data collection, data analysis or relationship between researchers and participants. | **Minor concerns:** In 24 of the 27 studies the body of data supported directly this review finding; and in one it was partial. | **Minor concerns:** The finding repeated across 26 studies. The strongest coherence was in studies reporting reasons of women requesting an elective CS | **Minor concerns:** In 15 studies fears related to vaginal birth were among the main findings, in the rest it was mentioned. | **High confidence** |
| **Caesarean section has advantages** | **Minor concerns:** There were three studies (of 35 studies) with serious or major methodological limitations, nine studies with moderate concerns, 16 studies with minor methodological limitations and five studies had no concerns regarding description of sampling, data collection, data analysis or relationship between researchers and participants. | **Minor concerns:** Two studies were indirect and three were of partial relevance. | **Minor concerns:** The finding was common in the majority of the studies especially among studies presenting elective CS. Studies including women with past negative experiences and anxious or unconfident women were those where the strongest coherence was found. | **Moderate concerns**: The least data adequacy was found in seven studies; Moderate rich data was achieved in 12 and 15 studies had rich data supporting this finding. | **High confidence** |
| **Quality of care** | **Moderate concerns:** There was one study (out of 12 studies) with serious methodological limitations, two studies with moderate concerns, and eight studies with minor methodological limitations including limited description of sampling, data collection, data analysis or relationship between researchers and participants. | **Minor concerns:** All studies were of direct relevance. | **No concerns:** One study had moderate concerns while 11 studies did not show any concern. | **Minor concerns:** One study had low adequacy and two had moderate. The rest of the studies were of high adequacy, with rich data. | **High confidence** |
| **Vaginal birth is the natural way to give birth** | **Moderate concerns:**  There were three studies (out of 26 studies) with serious or major methodological limitations, eight studies with moderate concerns, 10 studies with minor methodological limitations and three had no concerns at all, in relation to description of sampling, data collection, data analysis or relationship between researchers and participants. | **Minor concerns:** One study was indirect relevance, one was partial and 22 studies were direct relevance. | **No concerns:** The finding was common. One study had serious concerns, one had minor, and the rest had no concerns at all. | **No concerns:** Four studies had low adequacy, while two had moderate, and for the rest the finding was highly adequate | **High confidence** |
| **Vaginal birth is an empowering experience** | **Moderate concerns:**  There were two studies (out of 15) with serious methodological limitations, five studies with moderate concerns, seven studies with minor methodological limitations and three had no concerns at all, in relation to description of sampling, data collection, data analysis or relationship between researchers and participants. | **Minor concerns:** One study was partial relevance, and the rest were direct relevance. | **Minor concerns:** One study had serious concerns, seven had moderate, and the rest had no concerns at all. | **Moderate concerns:** Two studies had low adequacy, while nine had moderate, and for the rest the finding was highly adequate. | **High confidence** |
| **Caesarean section is risky** | **Minor concerns:**  There were two studies (out of 17) with major methodological limitations, and five studies with moderate concerns, while the rest were minor concerns and two had no concerns at all. | **Minor concerns:** two study was indirect relevance, and 17 studies were direct relevance. | **Minor concerns:** Ten studies had moderate concerns, and the rest had no concerns at all. | **Moderate concerns:** Three studies had low adequacy, while eight had moderate, and for the rest the finding was highly adequate. | **High confidence** |
| **The good mother imperative**  **factors Rite** | **Minor concerns:**  There were three studies with serious methodological limitations, four studies with moderate concerns, while the rest (9) were minor or no concerns at all. | **Minor concerns:** 14 studies (out of 15) were direct relevance. | **Minor concerns:** Eight studies had moderate concerns, and the rest had no concerns at all. | **Minor concerns:** Five studies had low adequacy, while five had moderate, and for the rest (5) the finding was highly adequate. | **High confidence** |
| **Religion advocates towards vaginal birth** | **Minor concerns:**  There was two studies with moderate methodological limitations, while the rest (6) were minor or no concerns at all. | **Minor concerns:** Seven studies (out of 8) were direct relevance. | **Minor concerns:** Seven studies had no concerns at all. | **Minor concerns:** Two studies had moderate adequacy, while the rest (6) the finding was highly adequate. | **High confidence** |
| **CS has economic and social implications**  . | **Moderate concerns:**  There was one study with major methodological limitations, six with moderate, six with minor limitations and five had no concerns at all. | **Minor concerns:** All studies were direct relevance and one was partial. | **Minor concerns:** One study had minor concerns, while the rest had no concerns at all. | **Minor concerns:** Three studies had low adequacy, six were moderate and the rest (9) were highly adequate. | **High confidence** |
| **Women decision towards mode of birth involves struggling to protect their right to decide** | **Minor to moderate concerns:** 10 studies had minor to moderate concerns, and four had minor or not concerns al all including limited description of sampling, data collection, data analysis or relationship between researchers and participants. | **Minor concerns:** In one study the body of data was indirectly support, in two was partial and in 11 the fit between the body of evidence and phenomena of interest was direct. | **No concerns:**  The finding repeated across 13 studies with no concerns. The strongest coherence was in studies reporting involvement of women with previous CS. | **Minor concerns:** In one study the quantity of data was low, in six was moderate and in seven was high. | **High confidence** |
| **Decision towards mode of birth is the result of an informed decision agreement** | **Moderate concerns:** There was two studies with serious methodological limitations, five studies with moderate concerns, and 12 studies with minor concerns including limited description of sampling, data collection, data analysis or relationship between researchers and participants. | **Minor concerns:** In three studies the body of data was partially support and in 16 the fit between the body of evidence and phenomena of interest was direct. | **No concerns:**  The finding repeated across nineteen studies with no concerns. This finding was cogent whether women were deciding to have VD or CS. | **Minor concerns:** In one study the quantity of data was low, in nine was moderate and in nine it was high. | **High confidence** |
| **Mode of birth is a medical decision** | **Minor to moderate concerns:** There was one study with serious methodological limitations, five studies with moderate concerns, and 11 studies with minor concerns and one with no concerns including limited description of sampling, data collection, data analysis or relationship between researchers and participants. | **Minor concerns:** In two study the body of data was indirectly support and in three was partial, while in 13 studies the fit between the body of evidence and phenomena of interest was direct. | **No concerns:**  The finding repeated across 16 studies with no concerns. This finding was cogent independently HCP decision. | **Minor concerns:** In nine studies the quantity of data was moderate, and in eight it was high. | **High confidence** |
