# Supplementary material for: Do women prefer caesarean sections? A qualitative evidence synthesis of their views and experiences
Source: PLoS One. 2021 May 5;16(5):e0251072. doi: 10.1371/journal.pone.0251072 (PMC8099111; doi:10.1371/journal.pone.0251072)
Supplement: S5 Table — (DOCX) [file pone.0251072.s005.docx]

**S5 Table.** Supporting quotes for final themes, initial concepts and emergent themes.

| **Final Themes** | **Initial Concepts** | **Emergent themes** | **Supporting quotes** |
| --- | --- | --- | --- |
| **Women beliefs underlying preferences for caesarean section as mode of birth** | **Deep rooted fears regarding vaginal birth** | **Fear of pain and injuries** | I was saying during the labour, “Get the knife out.” I just wanted a healthy baby and to survive this experience, and I thought “What is wrong with me?” I had no idea it would hurt so much; it would be so painful. I was saying, “Freeze, freeze my spine, freeze my spine!” I was saying, “It is no joke.” I wanted them to freeze my spine now (25). |
|  |  | **Fear of uncertainty** | No one could predict the outcome of my labour, so I wish to avoid potential stress and anxiety and have a planned caesarean (44). |
|  |  | **Fear of losing control over the body** | Karin: I don’t want the risks associated with tearing, I don’t want to push and sweat and moan and swear, I don’t want to lie and pooh [defecate] in front of anyone (45). |
|  |  | **Fear that vaginal birth would impact the tightness of vagina** | More women want to do the c-section now. Because of the beauty of the perineum. Women think that the fetal head is large and that will enlarge their vagina. And get vaginal tears. It is not aesthetic. And it is not good for the sexuality neither (34). |
|  | **Caesarean section has advantages** | **As a way of being under control** | I know how it will be and also when it will happen. It’s not that it comes as an unpleasant surprise down the road that can cause complications for the baby and for me. That is the greatest incentive to make me want to have a planned caesarean section, that it is the predictability. I do not feel at all comfortable with not knowing when, and how my body will handle labour and I do not know how I would react? For me it feels safer to know that I am in the hands of a team with physicians, anesthesiologists and nurses. This is where it is going to happen during this given day and time. You would not wake up in the middle of the night and wonder what is going on and where you should go. It is more organized with a planned caesarian section (53). |
|  |  | **To plan day and time (for convenience)** | If they know the date they’re due to give birth, they can tailor their work to the very last hour and give a precise date of return (58). |
|  |  | **To plan day and time  (for beliefs)** | My husband wanted the baby to be born on either a Monday or Thursday, but not on a Saturday. He said a Monday child will be clever and smart and if a baby is a boy he will become a soldier. A Thursday child will be a strong child and easy to raise. A Saturday child will have a dominant fate which means that he or she will be stubborn and may become aggressive and parents will have problem in bringing them up. The baby was due around Thursday and because my doctor’s schedule was not too busy on Thursday, we chose a Thursday (50). |
|  |  | **Because it is a more civilized way to give birth.** | Caroline: You know I think we’ve progressed with modern medicine and it’s [a Caesarean] (45). |
|  |  | **Because everybody does it** | I asked the physician and he said that there is nothing major that happens to worry about, it is safe, everybody is doing it and there are no risks (27). |
|  |  | **It is on the best interests of the baby** | ‘‘I think the baby may be deprived of oxygen during a normal birth, which may have negative outcomes. A caesarean eliminates this risk.’’ (Neslihan, S13) ‘‘People around me advise me to have a caesarean in order to not put my baby at risk.’’ (Tugba, S14) (39). |
| **Healthcare systems factors underlying preferences for caesarean section as mode of birth** | **Quality of care** | **To avoid inadequate support or care during childbirth** | I felt so much humiliation and I became angry. The doctor was blaming me for the delay of the delivery and she was making erroneous comments (28). |
| **Women beliefs underlying preferences for vaginal birth** | **Vaginal birth is the natural way to give birth** | **Because natural is better** | I’m not going to be stupid about it, but you know I think it’s [birth] a pretty natural thing and if everything is going naturally there’s no point in putting ourselves in a foreign environment where one can only be stressed (. . .) because as I said I think it’s important for the child and that whole birth process is important for it, I think it’s important that it chooses when it wants to enter the world, um (*) and it’s natural, I mean it’s the way things are designed to be done you know. (Angela, 24 year old university student, first pregnancy) (56). |
|  |  | **It is good for mother’s and baby´s health** | (VBAC) I’ve just read so much and I guess to me my belief was that the caesarean was going to be worse off for me and the baby than it would be to have a natural birth. Physiologically things would be better for us if we had a natural birth than to have a caesarean so every time someone suggested caesarean I just thought about those sorts of things. Like the successes of babies who’d had caesareans and most of them are born quite well but there’s certain things that sometimes I guess like breast feeding issues and things like that that they don’t often tell you. It can be harder to do when you’ve had a caesarean and things like that. By doing the vaginal birth you were really giving your baby a better chance – certainly (47). |
|  |  | **It ensures a quick recovery** | Already having a small toddler, I wish to be able to come home and continue as “normally” as possible and not have an extended hospital stay or recovery process (44). |
|  | **Vaginal birth is an empowering experience** | **Feelings of empowerment is desirable** | I wanted to feel the pain of childbirth, to feel like a woman. I wanted to have an active and not passive attendance in the process (28). |
|  | **Caesarean section is risky** | **Cesarean section exposes to negative outcomes** | ‘‘A caesarean requires anaesthesia and closure with stitches. I’m worried about them. I haven’t wanted to have caesarean.’’ (Gamze, V4) ‘‘I prefer a normal birth to having a scar from a cut.’’ (Leyla Yavuz, S 24) ‘I’ve met women who have had caesareans. They always complain. When they’re cold, they suffer from pain in their bellies. I’ve heard that I have to be careful with stitches, that I may have belly fat and that I shouldn’t become pregnant shortly after the surgery, etc.’’ (Banu, S8)(39). |
|  |  | **Cesarean section has sequalae and problems** | sutures will leave scars and cesarean sutures heal later and remain for a longer time compared to those of vaginal delivery. Besides, cesarean causes intrauterine adhesion, so you cannot have more than two cesareans because of that adhesion (51). |
| **Cultural factors affecting vaginal birth** | **The good mother imperative** | **Cesarean section is an easier and (lazy) alternative.** | Like my boss’s (*) niece had a baby and she had a caesar and there was no, medically she wasn’t (*) it wasn’t needed and I was, ‘Why would you have a caesar if you don’t need to?’ and then somebody started by saying, ‘Well, she’s too posh to push’ and I said ‘Oh my god’ (laughing) like hello, what are women there for? (Anke, 34 year old administrator, second pregnancy) (56). |
|  |  | **Good mothers have to sacrifice themselves** | Childbirth is messy. It is, you know? It's never pretty. That's a sacrifice you make, that's your rite of passage." She went on to link the idea of bodily sacrifice to fitness for motherhood: "If you're not ready to sacrifice your body for this child, then what are you really willing to sacrifice for this child, and why are you having children? ... I mean, why wouldn't you want to have your body go through whatever it has to [in order] to give birth?" p758 / "I would, [but] that's a horrible thing. Society is just like Women have to deliver vaginally, you're a better mom. ... You're failing if you do a C-section' (57). |
|  | **Religion advocates towards vaginal birth** | **Religious reasons** | Of course I’ll have a normal birth, because our prophet, Mohammed, recommended it. Eve also had a normal birth. However, let’s hope for the best.’’(Feride Kanat, S23) One woman even commented that natural birth helps one get rid of sins. ‘‘There is an idea that a woman who has a natural birth can get rid of her sins. It seems to be true . . . I always hear that prayers of women giving birth are accepted. (Begu¨m, V50)(39). |
|  | **Cesarean section has economic and social implications** | **Economic implications** | My husband said ‘if in this hospital they don't perform CS, I'm ready to pay a few million Tomans [the Iranian currency] to do CS in another hospital’. He also said ‘Even if I am forced to borrow money, I will not let you do NVD’ (W6) (59). |
|  |  | **Social implications: inability to fulfill women duties** | I’m saying that it would be a lot of hassle after the event, and being in a state with stitches or whatever and being told you can’t do this and you can’t do that for six weeks…my little boy’s at nursery and so it would be difficult if I can’t drive to get him to nursery and all that kind of thing (49). |
| **Women participation in power structures and decision making towards mode of birth** | **Women decision towards mode of birth involves struggling to protect their right to decide** | **Due to fear of going under an unnecessary CS** | Sally is the fourth provider I’ve seen during this pregnancy and I just started with her at 28 weeks! It took me that long to find a provider AND a hospital that would be supportive in this endeavor [VBAC]!! (April 08) (54). |
|  |  | **Due to fear of blame in the event of a poor outcome** | A: Well, the one who was there for my son’s birth, I felt like she abandoned us, so I didn’t appreciate her. She wasn’t trying any positioning things, nothing. She even played the dead baby card, which I thought only doctors did that! Q: What did she say? A: She was like, “Why are you willing to risk your baby’s life this way?” because we kept trying to get him out. So a few minutes after that, we were like, whatever. He’s not coming. So, we did the surgery (55). |
|  |  | **Strategies to maintain preferred mode of birth: homebirth** | Shame on the doctors for forcing me to choose between home birth and cesarean, when most agree that the safest place for me is to VBAC in a hospital with a supportive staff (54). |
|  | **Decision towards mode of birth is the result of an informed decision agreement** | **Continuity of care enables building confidence** | When you go to your GP and then you come to clinic and you have a different midwife and then you could see someone different on the team every time as well so for you to get, to build up some sort of confidence, to be talking to someone different every time and you are just repeating yourself. And you get to the stage where you are like what is the point in me telling you because you won’t see me the next time (33). |
|  |  | **Decision-making process by informed consent** | I have got a fantastic response at the antenatal clinic. I know that they understand. For them it was not a really big deal. It has been unbelievably good (53). |
|  | **Mode of birth is a medical decision** | **Women lack of autonomy regarding childbirth decisions** | Actually, she did not discussed it with me, she did not ask what I thought, I also did not ask. The cut [episiotomy] she also said that it is a thing to check in the moment, but we also did not discuss. I think that, I have a lot of confidence in her, what she had to do she was going to do it, you know? Then I also did not say if she wanted to cut or would not cut. This is something that I think the doctor have to decide, I think it’s something that we have not ... I’m completely lay, so I have not even an opinion, what is good, what is best , which is not. So I think if she spoke to me I would agree anyway (CAROLINA - typical patient) (67). |
|  |  | **Choice of mode of birth was health worker’s decision** | I think . . . it is not a question of . . . . I want to get rid of the decision making. I think that the decisions belong first of all to the health care providers, to the doctors, not the patients [Nora] (35). |
