# Supplementary material for: Do women prefer caesarean sections? A qualitative evidence synthesis of their views and experiences
Source: PLoS One. 2021 May 5;16(5):e0251072. doi: 10.1371/journal.pone.0251072 (PMC8099111; doi:10.1371/journal.pone.0251072)
Supplement: Attachment — Submitted filename: Response to reviewers.docx [file pone.0251072.s007.docx]

April 14, 2021

Dear academic editor,

Thank you for checking our manuscript.

As pointed out, Figure 2 was not referred to in the manuscript. A sentence was now included in line 305 for you to link the reader to the figure.

Thanks for your time and consideration. Looking forward to hearing from you,

Yours sincerely,

MSc. Mercedes Colomar

(Corresponding author)
